# Content-Sensing Based Resource Allocation for Delay-Sensitive VR Video Uploading in 5G H-CRAN

**DOI:** 10.3390/s19030697

**Published:** 2019-02-08

**Authors:** Junchao Yang, Jiangtao Luo, Feng Lin, Junxia Wang

**Affiliations:** 1 School of Communication and Information Engineering, Chongqing University of Posts and Telecommunications, Chongqing 400065, China; D150101004@stu.cqupt.edu.cn (J.Y.); D170101010@stu.cqupt.edu.cn (J.W.); 2 Electronic Information and Networking Research Institute, Chongqing University of Posts and Telecommunications, Chongqing 400065, China; Linfeng@cqupt.edu.cn

**Keywords:** VR video, content-sensing, resource allocation, delay-sensitive, 5G, H-CRAN

## Abstract

Virtual reality (VR) is emerging as one of key applications in future fifth-generation (5G) networks. Uploading VR video in 5G network is expected to boom in near future, as general consumers could generate high-quality VR videos with portable 360-degree cameras and are willing to share with others. Heterogeneous networks integrating with 5G cloud-radio access networks (H-CRAN) provides high transmission rate for VR video uploading. To address the motion characteristic of UE (User Equipments) and small cell feature of 5G H-CRAN, in this paper we proposed a content-sensing based resource allocation scheme for delay-sensitive VR video uploading in 5G H-CRAN, in which the source coding rate of uploading VR video is determined by the centralized RA scheduling. This scheme jointly optimizes g-NB group resource allocation, RHH/g-NB association, sub-channel assignment, power allocation, and tile encoding rate assignment as formulated in a mixed-integer nonlinear problem (MINLP). To solve the problem, a three stage algorithm is proposed. Dynamic g-NB group resource allocation is first performed according to the UE density of each group. Then, joint RRH/g-NB association, sub-channel allocation and power allocation is performed by an iterative process. Finally, encoding tile rate is assigned to optimize the target objective by adopting convex optimization toolbox. The simulation results show that our proposed algorithm ensures the total utility of system under the constraint of maximum transmission delay and power, which also with low complexity and faster convergence.

## 1. Introduction

In recent years, virtual reality (VR) technology has been rapidly commercialized, forming a $209 billion market by 2022 as predicted in [1]. VR makes use of 360-degree panoramic videos with high resolution (higher than 4K), high frame rate (60–90 fps) and low delay (less than 20 ms) to provide an immersive environment for the user to interact with the virtual world by a head-mounted display (HMD) [2]. Thanks to the development of VR display devices, the general public is able to experience VR capabilities on HMDs (e.g., HTC VIVE).

As the popularity of the User-Generated Content (UGC) platforms increases (e.g., Facebook and YouTube), more people would like to generate VR videos themselves with portable 360-degree cameras (e.g., GoPro OMNI, Samsung Gear 360, etc.) and share with others through UGC platform. Essentially, high-quality VR videos are produced and need to be uploaded to the UGC platform (uplink procedure); then, the UGC platform disseminates them to other VR viewers through applications like VR live broadcast (downlink procedure). These procedures, especially the uplink procedure, call for the use of 5G network as the strong support of mobility and collaboration. VR is predicted as the mass deployment of the 5G network [3], however, the transmission efficiency of VR video during those procedures in 5G network is one of the biggest issues in near future.

Optimization of VR video transmission is actively under explanation, but previous work mostly focused on the downlink procedure, and a common precondition of these studies is transcoding VR videos into multiple representations (bit rates) on the cloud server. Alface et al. [4] presented a typical tile-based adaptive downlink scheme which partitioned the panorama into independent tiles, enabling various content rate adaptable to the desired viewport of viewers. Recently, Corbillon et al. [5] presented another downlink adaptive streaming dependent on the predicted viewport and available bandwidth. As for the underlying technology, some researchers began to explore the potential of downlink delivering VR video over the cellular network (e.g., 5G ) by applying the multicast [6].

5G H-CRAN has been shown a promising solution for VR video transmission in the near future. Heterogeneous networks with massive densification of small cells and CRANs are combined in one network structure to improve spectral efficiency, resource management, and energy efficiency [7]. However, spectrum utilization and resource management are the major topics in H-CRAN [8]. Challenges and methodologies of RA in 5G H-CRAN has been reviewed in [9]. Recently joint RRH-association, sub-channel assignment and power allocation in multi-tier 5G H-CRANs has been studied in [10], which aimed at improving the system throughput. In addition, energy efficiency of nonorthogonal multiple access (NOMA) in C-RAN is researched in [11,12]. However, these researches mainly focus on downlink RA and power allocation (PA), which also fails to take into account the quality of content (QoC) [13] during RA.

This paper addresses the VR video uploading problem in 5G network. Optimization for VR video uploading in 5G network is more challenging and demanded. First, it is not viable for a user terminal to create multiple representations due to the lack of computing and storage resources on board. Second, the uplink wireless bandwidth is more limited than the downlink in 5G network. Third, content-sensing based source coding is necessarily adopted due to the high data volume of 360 degree content. Fourth, moving feature of UE which calls dynamic association with corresponding RRH/g-NB. The last not the least, delay-sensitive of VR video needs to be well scheduled in the uplink procedure.

Quite few studies VR video uploading over wireless network. However, RA for regular video uplink streaming over wireless network has been studied for different application types. Ragaleux et al. [14] presented an LTE uplink scheduling scheme for the heterogeneous QoS (Quality of Service) requirements of multimedia traffics, which was formulated as a joint time and frequency domain packet scheduling problem. El Essaili et al. [15] presented a systematic resource allocation and transmission optimization approach for the simultaneous streaming of user-generated video content. In particular, the centralized multi-user resource allocation problem and the distributed optimization of the video content at the mobile terminal were distinguished in the paper. In [16] surveillance video uplink streaming over wireless network was investigated, to solve the global video uplink streaming problem, they studied both the long-term bit-rate assignment for video encoding and the real-time packet scheduling in each OFDMA frame under the real-time constraint. Quality-of-Content (QoC) based joint source and channel coding in a mobile surveillance cloud has been investigated in [13], they aimed at optimizing the wireless resource usage so that more accurate human detections can be performed at the cloud server based on the received videos. To our best knowledge, VR video uplink streaming in 5G has not been researched in previous work, especially delay-sensitive VR video uploading under H-CRAN scenario.

Motivated by the aforementioned challenges for VR video uploading over wireless network, RA for delay-sensitive VR videos uploading in 5G H-CRAN has been studied in this paper. As the storage resources on board and delay-sensitive character of VR video, tile-based source coding [4] is adopt and only one bit rate representation for each video tile should be generated. Nevertheless, the source coding rate for each tile is bounded by maximum transmission delay and transmission rate of UE. Inspired by the previous work of QoC [13], we propose a content-sensing RA scheme for VR video transmission over 5G H-CRAN, which aiming at joint VR video source coding and uplink RA optimization. Instead of considering max-SINR [17] based RA in H-CRAN, in which consists of RRH association, PA and sub-channel allocation (SA) [18], the proposed scheme optimizes the total QoC which defined as weighted function of tile source coding rate. Then, we formulate the problem as an mixed-integer nonlinear problem (MINLP) [19].

Note that the problem is hardly solved in one shot by the optimization toolbox due to the problem consists of multiple stages in the VR video uploading process (i.e., RHH/g-NB association, sub-channel allocation and power allocation, and tile encoding rate assignment), a three stage algorithm is proposed to solve the problem efficiently in this paper. The total bandwidth is first allocated to g-NB groups according to the UE density of corresponding g-NB group, where frequency is reused at each RHH. Then RHH/g-NB association, sub-channel allocation and power allocation, and tile encoding rate assignment are jointly solved by decouple the problem into two sub-problem. First sub-problem can be described as allocate the optimal source coding rate for each tile under the constraint of maximum transition delay and upper bound of transmission rate, which is solved by optimization toolbox after the second sub-problem solved to obtain upper bound of transmission rate. While the second sub-problem can be expressed as find the optimization association, PA and SA to maximize the weighted sum-rate, which two step iterative algorithm is proposed to solve the sub-problem.

The remainder of the paper is organized as follows. System model and saliency of tile-based VR video are introduced in Section 2. The content-sensing based resource allocation scheme and problem formulation are described in Section 3. Our proposed algorithm for the problem is presented in Section 4. In Section 5, We evaluated the proposed scheme and algorithm by plenty of simulations. Finally, Section 6 concludes the paper.

## 2. System Model and Preliminary

### 2.1. System Model

**Figure 1:**
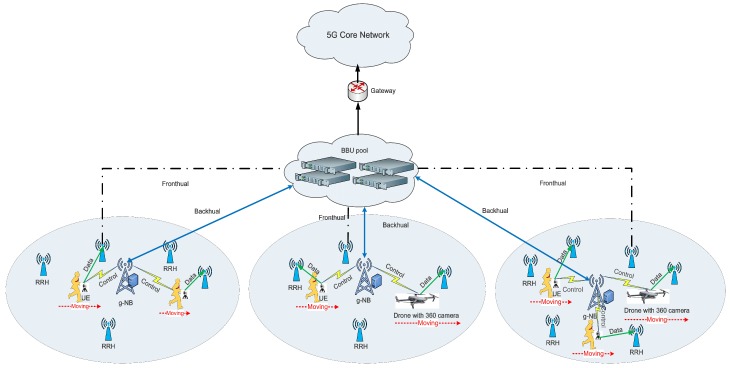
User generated VR video uploading in 5G H-CRAN.

The system considered in this work is depicted in Figure 1, where macro 5G base stations (i.e., g-NB) are underlaid with small cells (i.e., RRHs) [20], and the g-NBs and small cell RRHs are connected to a centralized BBU (baseband-unit) pool through backhaul links and fronthaul links, respectively. The BBU pool executes upper layer functions and baseband signal-processing, whereas, the RRHs normally perform as radio-frequency (RF) transceivers and only perform basic RF functions. SDN is adopted to support the separation of data and control information, which divides the control information into g-NB and data information into RRHs [7]. The g-NB is mainly responsible for the delivery of control information, and also serves UEs who are not associated with small cell RRHs. Moreover, cyclic prefix OFDM (i.e., CP-OFDM) [8] is considered as UL multiple access technique in our presented architecture.

Furthermore, consider *I* randomly User equipments (UEs) with 360-degree cameras which generate VR videos simultaneously, and need to upload them to the core network through 5G H-CRAN. Finally viewers experience the virtual reality in the HMDs by requesting the VR video contents through the UGC platform. Each VR video consists of multiple video chunks along the time which are denoted by z∈Z.

The RA scheduler is integrated with the BBU pool, and executes RA in the centralized approach. The available transmission bandwidth is subdivided into sub-channels, which are denoted by n∈N. These sub-channels are assigned in a centralized manner to each g-NB group which are denoted by g∈G. The set of assigned sub-channels to *g*-th g-NB group is denoted by $N^{g}$. The total sub-channels are allocated to each g-NB group disjointly. And the resource constraint is given in the following equation.

(1) $$\overset{G}{\underset{g}{\cup }}N^{g}\leq N$$

(2) $$N^{g}\cap N^{l}=\emptyset ,\forall g\neq l$$

To enhance the spectral efficiency, all the $N^{g}$ sub-channels are reused at each RRH and g-NB within the *g*-th g-NB group. On the other hand, UEs associated to a specific RRH within the coverage of the g-NB will share the $N^{g}$ sub-channels orthogonally (i.e., at a specific RRH, a sub-channel can be assigned to one of its associated UEs at a time) [9]. In addition, it is supposed that each UE is served by a single RRH or g-NB. For the simplicity of index, the RRHs and g-NB are uniformly denoted by m∈M. The resource constraint of *m*-th RRH/g-NB is expressed as follows

(3) $$n^{m∢g}\in N^{g},\forall g\in G,\forall m\in M,\forall n\in N\}$$

For the clarity of expression, we add upper index m∢g on sub-channel index *n* to denote that the reused sub-channel at RRH/g-NB *m* belonging to the assigned sub-channels of *g*-th g-NB group.

### 2.2. Tile Based Encoding and Saliency

Tile based encoding is widely adopted in the adaptive VR video streaming. With the motion-constrained tile sets (MCTS) coding, which has been introduced with the High Efficiency Video Coding (HEVC) codec, A VR video is spatially split into rectangular, independently decodable, non-overlapping regions after Equirectangular Projection (ERP). MCTS introduces a bit-rate overhead due to losses in compression efficiency [21]. However, tiling scheme allows more flexible adaptive streaming, especially in the context of omnidirectional videos where only a small portion of the content is displayed to the viewer at each instant time.

**Figure 2:**
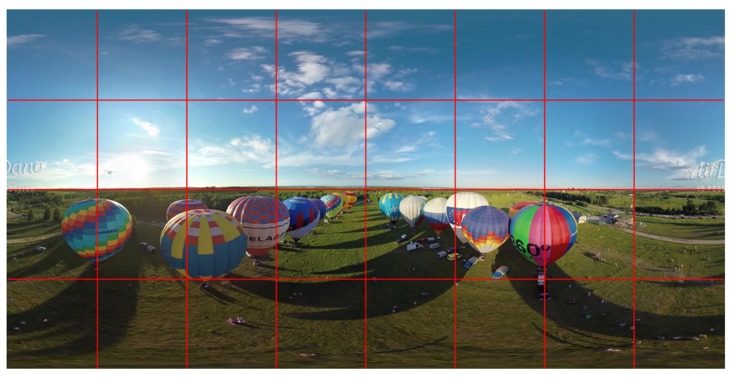
An ERP VR video frame with 4 × 8 tiles.

**Figure 3:**
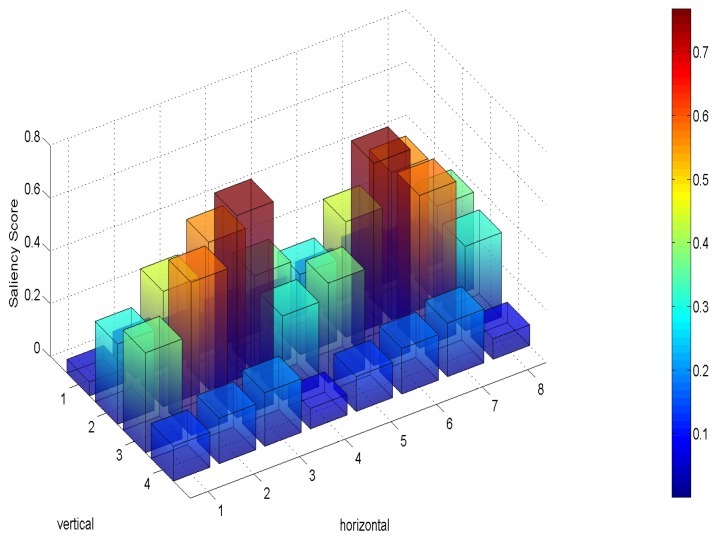
Saliency score of an ERP VR video frame with 4 × 8 tiles.

Saliency can be predicted using models of visual features, such as color, intensity and object. Figure 2 and Figure 3 show an ERP VR video frame with 4×8 tiles and the saliency score map by applying the saliency detection, respectively. Tiles with high saliency scores represent regions with attractive texture or object, to which a viewer would pay more attention. Distortions happened in a more salient region result in a much lower subjective quality scores of a perceived video [22]. Therefore, the viewer expect clear details (i.e., higher data rate) on the salient region. Additionally, as reported in [23] the viewer fixation in a 360-degree video is more preferred on the salient regions. Hence, the tiles with higher saliency score need higher bit rate to fulfill the desired perceived quality.

### 2.3. Utility Model

The QoE (Quality of Experience) metric for video networking quality evaluation is commonly used as Equation (4) defined in [24], where *U* denotes the utility of the video, *R* denotes the video rate, and α and β denote the coefficients of the utility model. $R_{M}$ represents the maximum video rate that Dynamic Adaptive Streaming over HTTP (DASH) server could provide, and let $R\in \{R_{1},\ldots ,R_{M}\}$, where $R_{1}$ and $R_{M}$ represents the minimum and maximum rate, respectively.

(4) $$U\left(R\right)=\left\{\begin{array}{c} \alpha \log(\beta R/R_{M}),R>0\\ 0,R=0 \end{array}\right.$$

It is well known that the encoding tile bit rate critically determine the perceived quality of VR content (i.e., QoC). Furthermore, for each tile, the saliency can also affect the perceived QoC. Inspired the QoE metric for downlink adaptive streaming, QoC metric for VR video uploading is introduced in this paper, which reflects the perceived quality of VR video after the source coding. Similarly, we defines the utility of one tile as (5), where $w_{i,j,z}$ denotes the weight of a tile to the QoC, and is defined as the saliency score of the tile. The utility of a VR video at *z*-th chunk can be defined as (6), where $R_{i,j,z}^{q}$ and $R^{Q}$ represent the encoding rate of *j*-th tile in *z*-th video chunk of *i*-th UE and the pre-defined maximum tile encoding rate, respectively. The QoC metric is strictly concave function of tile encoding rate, i.e., utility increase as tile encoding rate improve. And it also marginally decreasing [25]. Note that during the uplink procedure only one bit rate representation will be generated for each tile according to our proposed scheme in this paper. And traditional transcoding procedure can be adopted if needed for further downlink adaptive streaming.

(5) $$U_{i,j,z}=\left\{\begin{array}{c} w_{i,j,z}\cdot \alpha \log\left(\beta R_{i,j,z}^{q}/R^{Q}\right),R_{i,j,z}^{q}>0\\ 0,R_{i,j,z}^{q}=0 \end{array}\right.$$

(6) $$U_{i,z}=\sum_{j}^{J}U_{i,j,z}$$

The symbols and notations used in the paper are summarized in Table 1.

## 3. Content-Sensing RA Scheme and Problem Formulation

### 3.1. Content-Sensing RA Scheme

Different density of UEs within each g-NB (i.e., different resource requirement in each g-NB group) and moving characteristic of UEs lead unstable topology of the system, which calls for a centralized RA scheduler to dynamically allocate the resource. Furthermore, tiles in the same VR video probably need different bit rate to achieve reasonable perceived quality, while the source coding rate for each tile is bounded by the maximum transmission delay and transmission rate of UE. Essentially, the content in different tile region should be taken into consideration during the RA.

**Figure 4:**
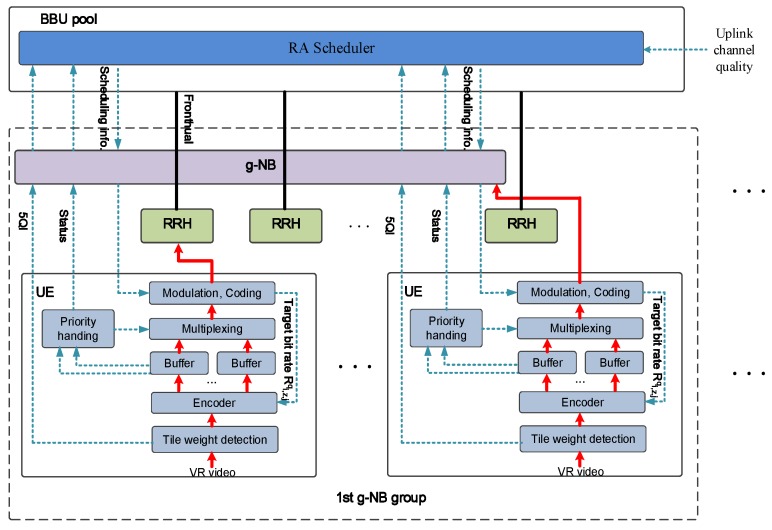
Diagram of Proposed Scheme.

Motivated by this, we proposed a novel Content-Sensing RA scheme for VR video uploading in 5G H-CRAN, which is depicted as Figure 4. RA scheduler integrated at the BBU pool dynamically allocate the resource to each g-NB group according to resource requirement of UEs within the g-NB group, and associate the UEs to g-NB or RRH based on the reported channel quality. The target of RA and UEs association is to maximize the total utility of VR video (i.e., QoC) under the constraint of resource, maximum transmission delay of video chunk and maximum UE power.

Each g-NB group is consisted by g-NB and RHHs under coverage of corresponding g-NB, which serve the UEs within the coverage of g-NB. Furthermore, all UEs report the channel quality to centralized RA scheduler through the g-NB (i.e., RA scheduler integrated with the BBU pool has the full knowledge of the channel side information), and associated to the corresponding RRH or g-NB according to the scheduling results.

For the UE part, once the VR video is generated, the saliency detection is executed every RA round by the tile weight detection module. Then the weight for each tile region is then quantized into the range of the 5G Quality of Service (QoS) class identifier (5QI), which is expressed as (7). Finally the weight is reported to the RA Scheduler through the g-NB.

(7) $$w_{i,j,z}\in \left\{1,\ldots ,K\right\},\forall i\in I,\forall j\in J,\forall z\in Z$$

Meanwhile, the RA scheduler extracts the quantized weight for performing RA in our scheme instead of ensuring bearer traffic’s QoS. Finally, the encoder performs the tile-based encoding, while each tile is encoded at the target bit rate according to the RA results. After the multiplexing and modulation and coding, the encoded video signal then is transmit to the BBU pool for upper layer functions and baseband signal-processing through the corresponding RRH or g-NB.

Note that RA scheduler integrated at the BBU pool play a key role in the proposed scheme. G-NB group RA, RHH/g-NB association, SA and PA, and tile encoding rate assignment are centralized determined by the scheduling results. In order to obtain a optimal solution for the RA scheduler, mathematical problem formulation and a three stage algorithm to solve the problem will be described in the following part.

### 3.2. Problem Formulation

According the aforementioned analysis, the objective is to maximize the total QoC for VR uploading under constraints of resource, transmission delay of video chunk and UE power, while source coding rate for each tile is bounded by the maximum transmission delay and transmission rate of UE. Therefore, the transmission rate and transmission delay of UE is investigated in the following:

The signal to interference and noise ratio (SINR) for *i*-th UE at *z*-th video chunk associated with *m*-th RRH on *n*-th sub-channel is calculated as follows [24].

(8) $$\gamma_{i,z}^{(m),n}=\frac{p_{i,z}^{(m),n}{\left|h_{i,z}^{(m),n}\right|}^{2}}{\sigma^{2}+I_{i,z}^{(m),n}}$$

where

(9) $$I_{i,z}^{(m),n}=\sum_{k\neq i}p_{k,z}^{(m),n}{\left|h_{k,z}^{(m),n}\right|}^{2}$$

The achievable data-rate of *i*-th UE at *z*-th video chunk when associated with *m*-th RRH/g-NB can be written as follows

(10) $$r_{i,z}^{\left(m\right)}=\sum_{n}^{N^{g}}x_{i,z}^{(m),n}W\log_{2}\left(1+\gamma_{i,z}^{(m),n}\right)$$

where $x_{i,z}^{(m),n}=1$ indicates *n*-th sub-channel on *m*-th RRH/g-NB assigned to *i*-th UE at *z*-th video chunk, and 0 otherwise. Note that a UE can be only associated with one RRH or g-NB at a certain time, the association can be changed as the motion of UEs. However the simplicity of system model and it is supposed that the association is unchanged during the period of each video chunk transmission.

The transmission delay for *i*-th UE of *z*-th video chunk can be calculated as

(11) $$d_{i,z}=\frac{B_{i,z}}{r_{i,z}}$$

where $B_{i,z}$ is the size of *z*-th video chunk of *i*-th UE, which can be written as (12), and *T* is the length of a video chunk, which is a fixed time length in this paper. And $r_{i,z}$ is the transmission rate of *i*-th UE at *z*-th video chunk, which is expressed as Equation (13), $y_{i,z}^{\left(m\right)}$ is introduced to indicate that *i*-th UE at *z*-th video chunk is served by *m*-th RRH/g-NB, $y_{i,z}^{\left(m\right)}$=1, and 0 otherwise.

(12) $$B_{i,z}=T\sum_{j}^{J}R_{i,j,z}^{q}$$

(13) $$r_{i,z}=\sum_{m}^{M}y_{i,z}^{\left(m\right)}r_{i,z}^{\left(m\right)}$$

The transmission power for *i*-th UE at *z*-th video chunk can be defined as

(14) $$p_{i,z}=\sum_{m}^{M}y_{i,z}^{\left(m\right)}\sum_{n\in N_{i,z}}p_{i,z}^{(m),n},\forall i\in I,\forall z\in Z$$

Thus the RA problem with the objective of total VR video utility maximization in the uplink of 5G H-CRAN subjected to the total resource constraint, maximum transmission delay of each video chunk and UE power constraint can be formulated as follows:

$$\begin{array}{l} OPT-1:\\ \underset{R_{i,j,z}^{q},y_{i,z}^{\left(m\right)},x_{i,z}^{\left(m\right),n^{m∢g}},N^{g}}{Max}\sum_{i}^{I}\sum_{j}^{J}\sum_{z}^{Z}U_{i,j,z}\left(w_{i,j,z},R_{i,j,z}^{q}\right)\\ s.t.\\ R_{i,j,z}^{q}\in \left\{R^{1},\ldots ,R^{Q}\right\},\forall i\in I,\forall z\in Z,\forall j\in J\left(C1\right)\\ d_{i,z}\leq d_{\max},\forall i\in I,\forall z\in Z\left(C2\right)\\ p_{i,z}\leq p_{i}^{\max},\forall i\in I,\forall z\in Z\left(C3\right)\\ y_{i,z}^{\left(m\right)}\in \left\{0,1\right\},\forall i\in I,\forall z\in Z,\forall m\in M\left(C4\right)\\ x_{i,z}^{\left(m\right),n^{m∢g}}\in \left\{0,1\right\},\forall i\in I,\forall z\in Z,\forall m\in M,\forall g\in G\left(C5\right)\\ n^{m∢g}\in N^{g},\forall m\in M,\forall g\in G\left(C6\right)\\ N^{g}\cap N^{l}=\emptyset ,\forall g\neq l\left(C7\right)\\ \overset{G}{\underset{g}{\cup }}N^{g}\leq N,\forall g\in G\left(C8\right) \end{array}$$

The constraint C1 limits target source coding rate of each tile is selected in the set of pre-defined tile encoding rate. C2 indicates that transmission delay for each video chunk should be less than the maximum delay due to limitation of storage in board and timeliness of VR video. C3 represents maximum per UE transmit power constraint. The constraint C4 indicates a UE can be served by a single RRH or the g-NB. C5 is constraint to ensure that n-th sub-channel is only allocated to one UE when the UE associated with RRH or g-NB. The C5 and C6 limit allocation sub-channel to UEs is in the assigned sub-channels set of corresponding g-NB group. The resource constraints C7 and C8 indicate assigned sub-channels set to each g-NB group is disjointed and sum of these sets is under the total resource constraint, respectively.

## 4. Algorithm for Problem Solution

Note that OPT-1 is mixed-integer nonlinear problem (MINLP) [19], However the optimization consists of g-NB resource allocation (i.e., $N^{g}$), RHH/g-NB association (i.e., $y_{i,z}^{\left(m\right)}$ ), SA (i.e., $x_{i,z}^{(m),n}$ ) and PA (i.e., $p_{i,z}^{\left(m\right)}$ ), and tile encoding rate assignment (i.e., $R_{i,j,z}^{q}$). Furthermore, Even for each separated sub-problem still needs sophisticated algorithm to reach optimal solution [18]. Therefore, the problem can be hardly solved by the existing methods and optimization toolbox.

**Figure 5:**
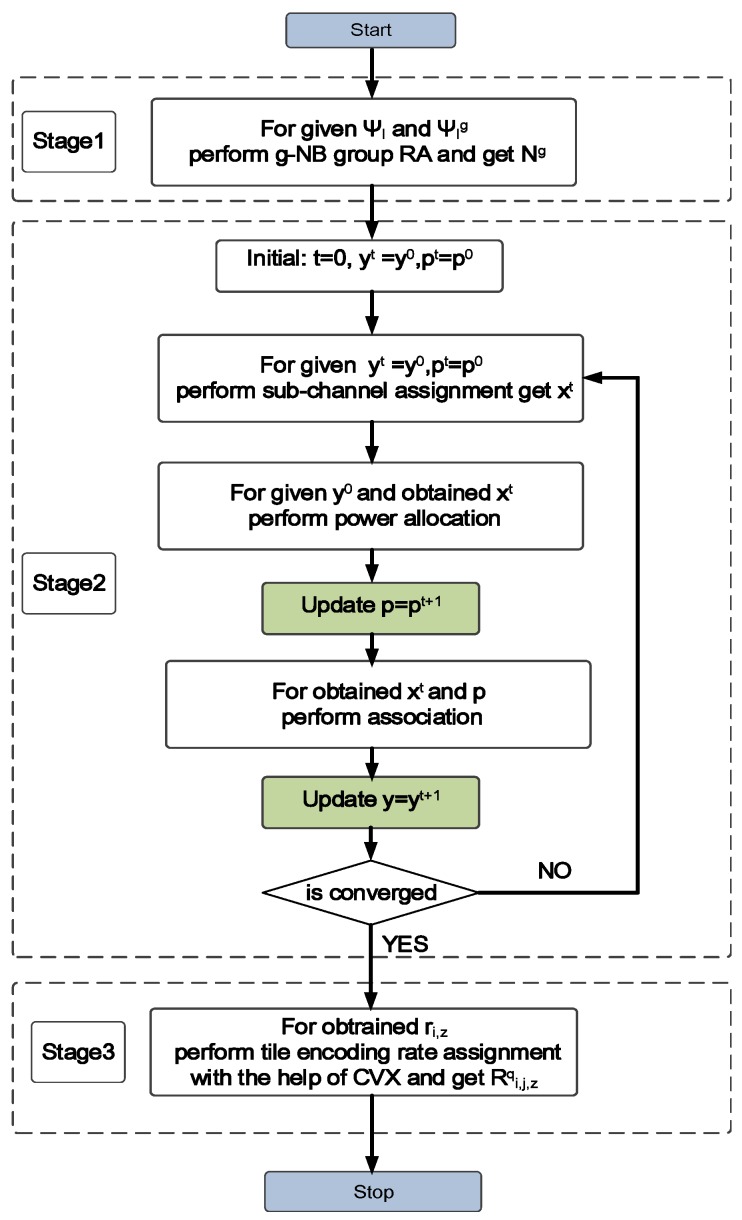
Flow chat of Proposed Algorithm.

In this paper, we propose a three-stage optimization algorithm to solve the problem. The flow chat of proposed algorithm is shown as Figure 5. Specifically, in stage 1, the total bandwidth is first allocated to cardinality of g-NB group according to UE density of corresponding g-NB group. Then RHH/g-NB association, SA and PA, and tile encoding rate assignment are jointly solved by decouple the problem into two sub-problem. First sub-problem can be described as allocate the optimal source coding rate for each tile under the constraint of maximum transition delay and upper bound of transmission rate, which is solved by optimization toolbox after the second sub-problem solved to obtain upper bound of transmission rate (which is shown as stage 3 in the flow chat). While the second sub-problem can be expressed as find the optimization association, PA and SA to maximize the weighted sum-rate, which two step iterative algorithm is proposed to solve the sub-problem (which is shown as stage 2 in the flow chat). And the detail of each stage of proposed algorithm will be described in the following subsections.

### 4.1. G-NB Group Resource Allocation

Note that total resource bandwidth is allocated to each g-NB group non-overlapping in order to mitigate the inter macro cell (i.e., g-NB) interference and protection of control information signal. However the sub-channels assigned to each g-NB group, which not only can be reused by the g-NB, but also the RRHs within the coverage of the g-NB can be reused these sub-channels. And our strategy is to dynamically allocate the resource according to the requirement. Essentially, the g-NB group resource allocation which should be determined by the number of RRH and the UE transmission requirement in each g-NB group. For the simplicity, in our system, we assume the same density of RHH within each g-NB (i.e., the same number of RHH in each g-NB group). Based on this assumption, the g-NB group RA is based on density of UEs within corresponding group, which can be expressed as

(15) $$\frac{N^{g}}{N}=\frac{\psi_{I}^{g}}{\psi_{I}}$$

(16) $$N^{g}\cap N^{l}=\emptyset ,\forall g\neq l$$

(17) $$\overset{G}{\underset{g}{\cup }}N^{g}\leq N,\forall g\in G$$

where $\psi_{I}^{g}$ and $\psi_{I}$ denotes number of UE within *g*-th g-NB group and total number of UE in the system.

Once g-NB group resource allocation completed, the OPT-1 problem can be rewritten as

$$\begin{array}{l} OPT-2:\\ \underset{R_{i,j,z}^{q},y_{i,z}^{\left(m\right)},x_{i,z}^{\left(m\right),n^{m∢g}}}{Max}\sum_{i}^{I}\sum_{j}^{J}\sum_{z}^{Z}U_{i,j,z}\left(w_{i,j,z},R_{i,j,z}^{q}\right)\\ s.t.\\ C1,C2,C3,C4,C5,C6 \end{array}$$

The OPT-2 can be described as joint RHH/g-NB association, SA, PA and source coding optimization problem. Notice that source coding rate of each tile is determined by tile weight (i.e., saliency) and upper bound of transmission rate of UEs. When transmission delay for each video chunk is fixed (i.e., equal to the maximum value), it is obvious the objective function could get optimal value only if each UE reaches the maximum transmission rate. Consequently, The OPT-2 problem can be rewritten as

$$\begin{array}{l} OPT-3:\\ \underset{R_{i,j,z}^{q}}{Max}\sum_{i}^{I}\sum_{j}^{J}\sum_{z}^{Z}U_{i,j,z}\left(w_{i,j,z},R_{i,j,z}^{q}\right)\\ s.t.\\ C1,C2\\ T\sum_{j}^{J}R_{i,j,z}^{q}\leq \bar{r_{i,z}}\times d_{max} \end{array}$$

where $\bar{r_{i,z}}$ represents the upper bound of the transmission rate of *i*-th UE at *z*-th chunk.

Note that OPT-3 is an convex problem if the $\bar{r_{i,z}}$ is known. In addition, OPT-3 can obtain the optimal solutions only if all the transmission rate of UEs reach the maximum. Consider the weighted sum-rate fairness of the system [18], upper bound of the transmission rate for each UE can be obtained by solving OPT-4 problem. Finally, joint RHH/g-NB association, sub-channel allocation and power allocation, and tile encoding rate assignment optimization problem are decoupled into two sub-problem (i.e., OPT-3 and OPT-4).

$$\begin{array}{l} OPT-4:\\ \underset{y_{i,z}^{\left(m\right)},x_{i,z}^{\left(m\right),n^{m∢g}}}{Max}\sum_{i}^{I}\sum_{z}^{Z}w_{i,z}\bar{r_{i,z}}\\ s.t.\\ C3,C4,C5,C6 \end{array}$$

where $w_{i,z}=\sum_{j}w_{i,j,z}$, denotes the total tile weight of *z*-th chunk of UE *i*. Note OPT-4 can be described as an Joint RRH/g-NB association, SA and PA problem and OPT-3 can be expressed as an tile encoding rate assignment problem under the constraint of upper bound transmission rate. And Stage 2 and Satge 3 of proposed algorithm solve the OPT-4 and OPT-3, respectively, which details are explained in the following part.

### 4.2. Sub-Channel Allocation and Power Allocation

The OPT-4 can be described as an weighted sum-rate maximization problem, and given the fixed association, the problem has be proven an NP-hard problem in [26,27]. Furthermore, the OPT-4 is also a MINLP. Even for a given RRH/g-NB association and PA, Find the optimal SA among the UEs alone is difficult due to the large search space of the optimization. Consequently, exhaustive search is not practical to solve the OPT-4. However, a two-step iterative algorithm is proposed in the Stage 3 which jointly optimizing the RRH/g-NB association, SA and PA. The basic idea is that SA and PA are performed with fixed association in first step while association is updated in second step. And the detail is discussed in the following subsections.

#### 4.2.1. Sub-Channel Allocation

For an initial RRH/g-NB association $y^{0}$ and initial PA $p^{0}$, the SA problem can be rewritten as

$$\begin{array}{l} OPT-5:\\ \underset{x_{i,z}^{\left(m\right),n^{m∢g}}}{Max}\sum_{i}^{I}\sum_{z}^{Z}w_{i,z}\bar{r_{i,z}}\\ s.t.\\ C5,C6 \end{array}$$

For initialization, we employed path-loss based association and uniform PA. More specifically, for RRH association, $y_{i,z}^{\left(\bar{m}\right)}=1$ for $\bar{m}=\arg\min\xi_{i,z}^{m}$ and $\min\xi_{i,z}^{m}\leq \xi_{\max}$, otherwise 0; while $\min\xi_{i,z}^{m}\geq \xi_{\max}$, UE associates to the g-NB of corresponding group (i.e., $y_{i,z}^{\left(\bar{m^{\prime }}\right)}=1$). Where $\xi_{i,z}^{m}$ represents the distance between UE *i* and RRH *m*, and $\xi_{\max}$ represents the predefined maximum distance between UE and RRH. $\bar{m}$ represents the initial association of RRH index, $\bar{m^{\prime }}$ represents the g-NB index of corresponding group which UE belongs. And the uniform PA $p^{0}$ can be expressed as follows

(18) $$p_{i,z}^{0}=(p_{i,z}^{\max}/\left|N^{g}\right|)$$

So that for the fixed RRH/g-NB association and PA in each g-NB group, the achievable rate of each UE on each sub-channel is calculated iteratively and then the sub-channel is assigned to the UE which having highest weighted achievable rate on that sub-channel in each iteration. Finally, all the sub-channels are assigned to UEs according to the (19). where $x^{t}$ is sub-channel assignment result of *t* iteration.

(19) $$x^{t}=\arg\max(w_{i,z}\bar{r_{i,z}})$$

#### 4.2.2. Power Allocation

In this step, PA is performed more precisely after the SA. Note that the initial PA is performed in an uniformed way. However, after getting the sub-channels assigned to each UE $N_{i,z}$ with the initial association, the PA can be reallocated across the $N_{i,z}$. Equal Power Distribution (EPD) and Interior Point Algorithm (IPA) are the two common way of PA. However the IPA is more attractive method for fast coverage and easy management of inequality constraint [10]. In addition, IPA provides optimal PA.

The IPA involves four phases to get optimality conditions. First, the inequality constraints are transformed into equality constraints by the addition of slack-variables to the former. Second, non-negativity situations are implicitly tackled by adding them to the objective function as logarithmic barrier terms. Third, the optimization problem with equality constraints is transformed into unconstrained optimization problem. Fourth, the perturbed Karush-Kuhn Tucker (KKT) first order optimality conditions are solved through the Newton method [19].

While EPD allocates the maximum transmission power of UE equally distributed among the assigned sub-channels of UE. And the maximum transmission power of UE *i* is set as $p_{i}^{\max}$, power can be allocated as follows

(20) $$p_{i,z}^{(m),n}=p_{i}^{\max}/\left|N_{i,z}\right|$$

Note the EPD is also an alternative method with the simplicity and low complexity compared with the IPA. Consequently, the PA solution is updated as $p^{t+1}$ after PA. In addition, performances of two PA method will be compared in the simulation part.

#### 4.2.3. RRH/g-NB Association

Since the initial association is based on path-loss, which may not be the optimal association. RRH/g-NB association optimization is performed in this step. After SA and PA, we try to optimize RRH/g-NB association with x and $p^{t+1}$, which can be formulated as

$$\begin{array}{l} OPT-6:\\ \underset{y_{i,z}^{\left(m\right)}}{Max}\sum_{i}^{I}\sum_{z}^{Z}w_{i,z}\bar{r_{i,z}}\\ s.t.\\ C4 \end{array}$$

Due to sub-channels are reused at each RRH within corresponding g-NB group, RRH/g-NB association can be performed with x and $p^{t+1}$ to reach optimal association. Note that OPT-6 can be solved by setting all $y_{i,z}^{\left(m\right)}$ to zeros except that $y_{i,z}^{\left(\bar{\bar{m}}\right)}=1$, where $\bar{\bar{m}}$ represent the association which achieve the best weighted achievable rate of UE which is given in (21). And the problem can be solved step-by-step by setting the $y_{i,z}^{\left(m\right)}$ =1 until all the UEs are associated with RRH/g-NB.

(21) $$\bar{\bar{m}}\;=\arg\max(w_{i,z}\bar{r_{i,z}})$$

**Figure 6:**
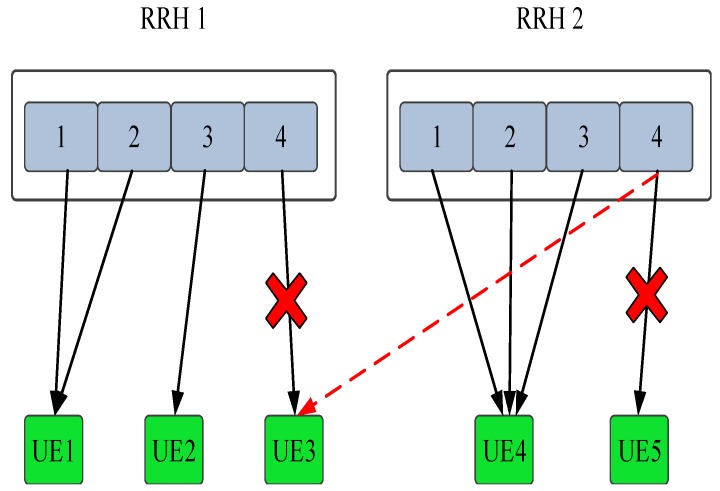
Diagram of an association example.

One thing need to point out that method in [10] to solve the problem by relaxing the binary constrain to continuous, which may solves the problem, however it conflicts with the real practice that one UE is only associated with one RRH/g-NB, which may also leads the sub-optimal solutions. In our proposed method, we just need to calculate the weighted achievable rate of UE across the g-NB group with the assigned sub-channels and power. Then choose the best association based on the weight achievable rate. The detail can be demonstrated as the Figure 6 with given simple example. More specifically, the solid arrow line indicates the initial association and sub-channel allocation. UE 3 initially associated with the RRH 1 and the sub-channel 4 is assigned to it. However, the weighted achievable rate of UE 3 on the sub-channel 4 of RRH 2 is much higher than the initial association, hence, the association of UE 3 is changing from RRH 1 to RRH 2 according to our strategy.

Convergence of stage 2 is judged by comparing the previous iteration association with the current association. If the association is unchanged among all UEs, which indicates all UEs associate the optimal RRH/g-NB. Otherwise, the algorithm perform SA and PA again with current RRH/g-NB association (i.e., $y^{t+1}$ ).

### 4.3. Tile Encoding Rate Assignment

The upper bound transmission rate of UE can be obtained once association and sub-channel allocation completed. For given upper bound transmission rate of UE, the sub-problem OPT-3 can be expressed as assign optimal encoding rate for each tile of z-th chunk of UE. Note that the OPT-3 is an convex problem after relaxation of discrete tile encoding rate to continuous. And the problem can be solved by the convex optimization toolbox (e.g., CVX), the similar problem has been studied in our previous work.

## 5. Simulation

### 5.1. Setup

There g-NB cell with 1 km radius is considered in the simulation, where two RRHs is uniformly distributed within g-NB cell. The users’ locations are randomly generated and UE follow a Poisson Point Process (PPP) with density $\lambda_{g}$ distributed in each g-NB group. The maximum transmit power of each UE is 23 dBm and the system bandwidth is 100 MHz consisting of *N* orthogonal sub-channels. ITU pedestrian B fast fading model and the COST231 Hata propagation model for micro-cell environment [26] are adopted. Lognormal shadowing with 8 dB standard deviation is implemented. The noise power spectral density is assumed to be −173 dBm/Hz.

The predefined minimum and maximum tile encoding rate is set $R^{1}=0.5$ Mbps and $R^{Q}=5$ Mbps, respectively. And the maximum transmission delay is set as 1 second. Furthermore, coefficients of the utility model α and β is empirically set 0.1 and 1000, respectively. The length of one video chunk is set as 1 s in the simulation.

For performance comparison, different combination of schemes in each stage of algorithm is evaluated, which are summarized as follows:

- Fixed g-NB group resource allocation (FGRA) + IPA power allocation (IPA) + Proposed RRH/g-NB association (PAS) + Tile rate assignment with CVX (TRAC): The group resource allocation in stage 1 is fixed, and will not change when the UE density in each group changed as the motion of UEs (i.e., VR video transmission requirement is different in each g-NB Group). And each group allocate the fixed resource to UEs of the group in a decentralized way. And PA and SA is performed according to IPA and PAS respectively. In stage 3, the tile rate assignment is according the solution of relaxed convex problem.
- Dynamic g-NB group resource allocation (DGRA) + IPA + PAS + TRAC: The group resource allocation is dynamically based on the UE density within each group. IPA and PAS is adopted in stage 2. And TRAC is performed in stage 3.
- DGRA + EPD + PAS + TRAC: DGRA and PAS is performed in stage 1 and stage 2, while the power allocation follows EPD scheme. And TRAC is performed in stage 3.
- DGRA + IPA + PAS + Tile rate assignment with global search (TRAGS): stage 1 and stage 2 adopt the DGRA, IPA and PAS, respectively. however the tile rate assignment in stage 3 using the global search to obtain the optimal solution.
- DGRA + IPA + association as in reference [10] (ASR) + TRAC: DGRA and IPA is adopted in stage 1 and stage 2, however g-NB association performed according to the results for relaxation of association constraint.And TRAC is performed in stage 3.
- DGRA + IPA + path-loss based association (PLAS) + TRAC: DGRA and IPA is adopted in stage 1 and stage 2, however g-NB association performed based on the path-loss (i.e., UE associates the nearest RRH/g-NB). And TRAC is performed in stage 3.

### 5.2. Results

**Figure 7:**
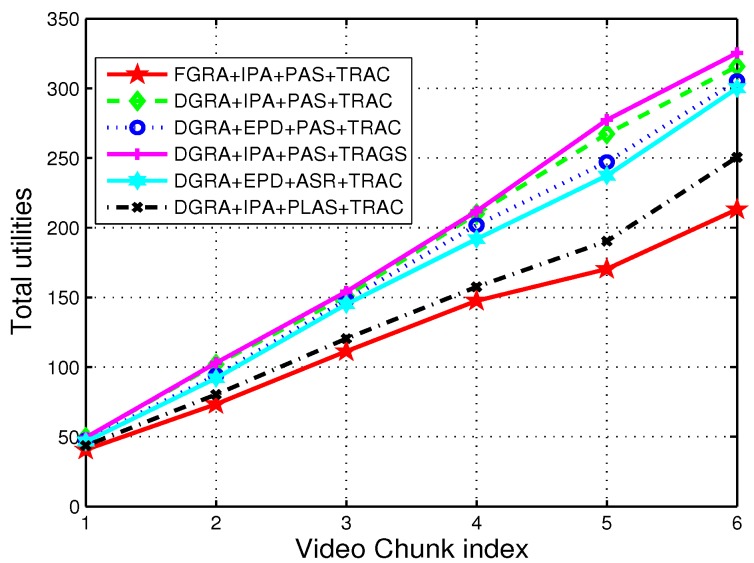
Total utility with different schemes combination.

Figure 7 shows total utility with different combination of schemes in each stage of the solution algorithm along with time (i.e., video chunk index in the Figure 7). it is clearly shows that the DGRA + IPA + PAS + TRAC and DGRA + IPA + PAS + TRAGS performs better than the other combinations. And the only difference between the two combinations is that tile rate assignment in stage 3 performed by the global search (i.e., optimal solution) and convex toolbox respectively. However, the TRAGS (i.e., global search) could achieve better than the TRAC (i.e., convex toolbox), the complexity is much higher than the TRAC shceme. Table 2 shows that CPU time of two different schemes under the same conditions (i.e., the preceding stages adopt DGRA + IPA + PAS to obtain the upper bound transmission rate $\bar{r_{i,z}}$ ), TRAC is four times faster than TRAGS, this is because TRAGS search all the possible solutions while TRAC using the approximation method to get the optimal solution. And We could see that the utility with TRAGS performs slightly better than that with TRAC. Therefore TRAC is an attractive scheme with less CPU time to obtain considerable utility compared to TRAGS.

Note that fixed resource for each g-NB group performs the worst (i.e., FGRA + IPA + PAS + TRAC), since it lost adaptive resource allocation among the groups at centralized BUU pool. In addition, RRH/g-NB association based on reference [10] (i.e., DGRA + IPA + ASR + TRAC) and path-loss (i.e., DGRA + IPA + PLAS + TRAC) perform not as good as our proposed association method (i.e., DGRA + IPA + PAS+ TRAC), since the PLAS is not the optimal solution for the association after the SA and PA; while the ASR conflicts with real practice that one UE is only associated with one RRH/g-NB, and which probably leads the sub-optimal solutions.

**Figure 8:**
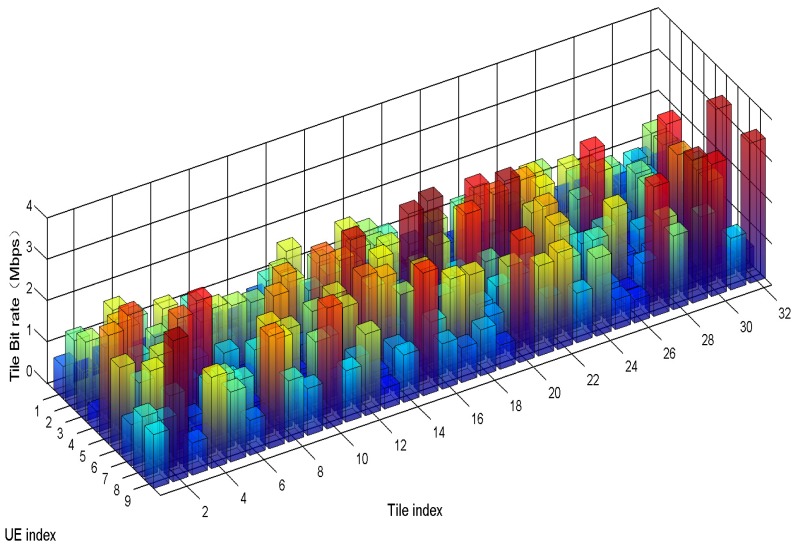
Tile encoding rate of each UE.

Figure 8 shows obtained optimal tile encoding bit rate for each UE with DGRA + IPA + PAS + TRAC. It can be seen that tile with higher saliency score is assigned higher bit rate, which is in accordance with the our motivation.

**Figure 9:**
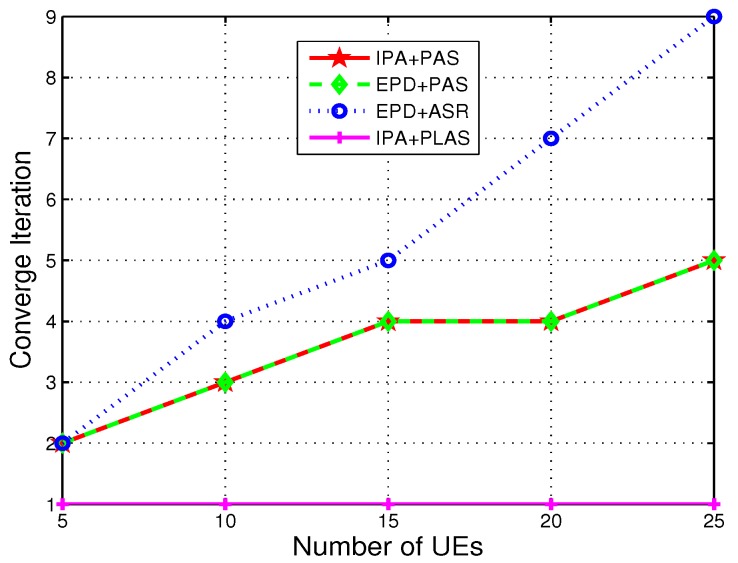
Iteration of different schemes in stage 2.

Figure 9 shows that converge iteration of different schemes in stage 2 as number of UEs increases. Notice that path-loss based association just need 1 iteration for converge, since each UE only need to associated the nearest RRH according to PLAS. In addition, in our simulation, if the nearest distance of UE to RRH is larger than predefined distance, it will associate the corresponding g-NB. The converge iteration of EPD + ASR is increased faster than that of EPD + PAS as the number of UEs increases. And the PAS will converge in several iterations. Note that convergence is not affected by the power allocation schemes, so that IPA + PAS and EPD + PAS perform the same iteration for converge with same UEs in the system.

Finally, The computational complexity of proposed algorithm is analyzed. Note that the proposed algorithm is consisted by five sub-stages (i.e., g-NB resource allocation, RRH/g-NB association, SA, PA and tile encoding rate assignment) to obtain optimal solution. For simplicity, we ignore the complexity of the first stage, which is typically very fast. In addition, the complexity of tile encoding rate assignment is also not discussed, however, the CPU time of two different schemes is already given in Table 2. Hence, complexity of different combination schemes of the solution algorithm is given in Table 3.

Where the T is the necessary number of iteration to convergence. Note that stage 2 is most computational part of the algorithm, which is mainly consisted of three parts (i.e., RA, PA, and association). Specifically, The SA complexity is order of number of sub-channels since all sub-channels are assigned to UE which having highest weighted achievable rate one by one; The complexity of PA with IPA and EPD is order of logarithm of number of sub-channels and order of number of sub-channels, respectively. For proposed association method (i.e., PAS), the complexity is order of number of UEs. The complexity of reference association method is order of number of RRHs power of number of UEs. while the path-loss based association can be performed at onetime.

Overall, proposed DGRA + IPA + PAS + TRAC is an attractive combination for the solution algorithm with better achievable utility, faster converge and less executive time.

## 6. Conclusions

In this paper, we studied delay-sensitive VR video uploading in 5G H-CRAN, and proposed an centralized scheme to allocate the resource based on content sensing, which is further formulated as a weighted utility optimization problem. A three stage algorithm is proposed to solve the MINLP problem. Dynamic g-NB group resource allocation is first performed according to the UE density of each group. In stage 2, joint RRH/g-NB association, sub-channel allocation and power allocation is performed by an iterative process.Finally, encoding tile rate is assigned to optimize the target objective with the help of convex optimization toolbox in stage 3. The simulation results show that the proposed algorithm with fast converge speed can achieve better utility (i.e., QoC), which is a promising solution for the MINLP problem.

## Figures and Tables

**Table 1:** Symbols and notations.

Symbol	Notation
I	Total number of UEs
i	Index of UE
J	Total number of tiles in a VR video frame
j	Index of tiles in a VR video frame
z	Index of VR video chunk
Z	Total number of chunk in one VR video
α, β	The coefficient of utility
$w_{i,j,z}$	The saliency weight of *j*-th tile at *z*-th chunk of *i*-th UE
$R_{i,j,z}^{q}$	The source coding rate of *j*-th tile at *z*-th chunk of *i*-th UE
U	Utility function
$d_{i,z}$	Transmission delay of *z*-th chunk of *i*-th UE
$B_{i,z}$	The size of *z*-th chunk of *i*-th VR video
T	The length of a video chunk
m	Index of RRH/g-NB
M	Total number of RRHs and g-NB
g	Index of g-NB group
G	Total number of g-NB groups
$r_{i,z}^{\left(m\right)}$	The transmission rate of *i*-th UE at *z*-th video chunk when associated with *m*-th RRH
$I_{i,z}^{(m),n}$	The interference on *n*-th sub-channel of *i*-th UE at *z*-th video chunk to *m*-th RRH/g-NB
$p_{i,z}^{(m),n}$	The transmit power of *i*-th UE at *z*-th video chunk to *m*-th RRH/g-NB
$N_{i,z}$	The set of sub-channels assigned to *i*-th UE at *z*-th video chunk to *m*-th RRH/g-NB
$|h_{i.z}^{(m),n}|$	The channel gain of *i*-th UE at *z*-th video chunk to *m*-th RRH/g-NB
N	Set of total sub-channels
$N^{g}$	Set of sub-channels assigned to g-th g-NB
$n^{g}$	Index of sub-channel of g-th g-NB group
$N^{m∢g}$	sub-channels set of *m*-th RRH under the coverage of g-th g-NB
$n^{m∢g}$	Index of sub-channel with *m*-th RRH/g-NB

**Table 2:** CPU times with different tile bit rate assignment schemes.

Schemes	CPU Time (s)
TRAC	0.859
TRAGS	3.015

**Table 3:** Complexity of different combination schemes of solution algorithm.

Schemes	Complexity
SA + IPA + PAS	$O(T\cdot \left(N^{g}+log\left(N^{g}\right)+I\right))$
SA + EPD + PAS	$O(T\cdot \left(N^{g}+N^{g}+I\right))$
SA + EPD + ASR	$O(T\cdot \left(N^{g}+N^{g}+M^{I}\right))$
SA + IPA + PLAS	$O(T\cdot \left(N^{g}+log\left(N^{g}\right)+1\right))$
